# Formulation and Stability Screening of Walnut Protein Concentrate-Whey Protein Isolate Stabilized Pickering Emulsions with Different Compounding Ratios: Capsanthin Loading, Multi-Condition Stability, and Application as a Butter Replacer in Spreadable Products

**DOI:** 10.3390/foods15193427

**Published:** 2026-09-25

**Authors:** Mihreban Sawaidul, Jiarui Li, Mingqiang Xu, Jian Zhang, Ting Zhang, Xintao Meng, Yan Ma

**Affiliations:** Institute of Agro-products Processing, Xinjiang Uygur Autonomous Region Academy of Agricultural Science, Research Center of Agricultural Products Processing Engineering, Urumqi 830091, China; 18196051811@163.com (M.S.); 18199846287@163.com (J.L.); xmq2046@hotmail.com (M.X.); zhangjian9666@126.com (J.Z.); zhangtingkikie@163.com (T.Z.); xintao-929@126.com (X.M.)

**Keywords:** Pickering emulsion, WPI-WalPC blending, capsanthin delivery, protein stabilization, butter substitute

## Abstract

This study developed a dual-protein Pickering emulsion stabilized by walnut protein concentrate (WalPC) and whey protein isolate (WPI) for capsanthin delivery and functional fat replacement. Pickering emulsions with different WPI-WalPC ratios were prepared, and their functional properties and stability were systematically evaluated. The results showed that the WPI-WalPC ratio of 1:9 exhibited the optimal performance, with the highest emulsifying activity index (EAI, 48.55 m^2^/g) and emulsifying stability index (ESI, 240.91 min), as well as superior stability under different environmental conditions. The enhanced stability was closely related to the formation of a robust protein-stabilized interface, which improved the protection and delivery efficiency of capsanthin. Compared with capsanthin directly incorporated into walnut oil, the optimized Pickering emulsion exhibited higher capsanthin retention under storage, light, thermal, and high-pressure treatments. Furthermore, replacing 30% of butter with the capsanthin-loaded Pickering emulsion in spreadable paste improved sensory acceptability and oxidative stability, accompanied by reduced acid and peroxide values. Overall, this study provides a promising strategy for constructing dual-protein Pickering emulsions as multifunctional carriers for lipophilic compounds and functional ingredients for developing nutritious spreadable foods.

## 1. Introduction

Proteins are considered ideal food-grade stabilizers for Pickering emulsions due to their excellent nutritional value, amphiphilic properties, and interfacial activity [1]. Whey protein isolate (WPI) exhibits excellent emulsifying ability [2]. Nevertheless, single-protein-stabilized emulsions may suffer from insufficient interfacial coverage and limited structural stability. The combination of proteins from different sources provides a potential strategy to overcome these limitations through complementary molecular interactions [3]. Walnut protein concentrate (WalPC), a plant-derived protein rich in functional groups and nutritional components, may interact synergistically with WPI to enhance interfacial adsorption and improve emulsion stability [4]. However, the effects of WPI/WalPC ratios on the emulsifying properties and stability of dual-protein Pickering emulsions remain unclear, and the optimal protein proportion for stable walnut oil-based Pickering emulsions requires systematic exploration.

Capsanthin, a natural carotenoid extracted from red pepper, is widely utilized as a natural food colorant with outstanding antioxidant activity [5]. Nevertheless, its practical industrial application is severely restricted by poor water solubility and high susceptibility to light, heat, and oxidative degradation [6,7]. Multiple delivery strategies, including inclusion complexes [8], microencapsulation [9], and lipid-based carriers [10], have been developed to improve the stability of lipophilic carotenoids. However, few studies have focused on the formulation of dual-protein Pickering emulsions using plant-animal protein combinations for capsanthin protection. Furthermore, the loading efficiency and stabilizing performance of such composite emulsions compared with direct oil incorporation remain poorly understood. Therefore, developing stable dual-protein Pickering emulsions provides a promising strategy to improve capsanthin stability and expand its applicability in functional food systems.

Therefore, this study aimed to construct a walnut oil-based Pickering emulsion stabilized by WPI-WalPC blends and investigate the effects of different protein ratios (0:10, 1:9, 3:7, 5:5, 7:3, and 9:1 (*w*/*w*)) on its functional properties and stability. The optimal WPI-WalPC ratio was selected based on comprehensive evaluation, and the resulting Pickering emulsion was subsequently used as a carrier for capsanthin. The loading performance of capsanthin-loaded Pickering emulsions, as well as their environmental stability were compared with that of capsanthin directly incorporated into walnut oil under environmental stresses (e.g., light, heat, and oxidative conditions). Finally, the optimized capsanthin-loaded Pickering emulsion was applied as a butter substitute in spreadable food products, and the effects of different substitution ratios on physicochemical, nutritional, and sensory qualities were evaluated. This study provides a novel strategy for developing dual-protein Pickering emulsions as multifunctional carriers for lipophilic pigments and functional fat-replacement ingredients.

## 2. Materials and Methods

### 2.1. Materials and Instruments

Walnut protein concentrate (protein content: 60%) was purchased from Peiling Biotechnology Co., Ltd. (Shandong, China); whey protein (purity ≥ 90%) was purchased from Qiyuan Biotechnology Co., Ltd. (Shandong, China), Walnut oil (food grade) was purchased from Xinjiang Aksu Shengding Oil Co., Ltd. (Aksu City, China); capsanthin (color value 245) was prepared in our laboratory. Pepsin (BR grade, activity 1:10,000 U/mg) and bile salt from pig (BR grade, cholic acid ≥ 65%) were purchased from Beijing Solarbio Science & Technology Co., Ltd. (Beijing, China). Isopropanol and sodium hydroxide (all analytical grade) were purchased from Tianjin Zhiyuan Chemical Reagent Co., Ltd. (Tianjin, China). N-hexane, ethanol, sodium chloride, anhydrous ethanol, and sodium hydroxide (all analytical grade) were purchased from Tianjin Zhiyuan Chemical Reagent Co., Ltd. (Tianjin, China). Sodium dodecyl sulfate (SDS, ACS reagent grade, ≥99.0%) was purchased from Sigma-Aldrich (St. Louis, MO, USA). Urea (molecular weight 60.06) and sodium dodecyl sulfate (SDS; molecular weight: 288.43; active substances ≥ 86%) were purchased from Xilong Science Co., Ltd. (Guangzhou, China). Distilled water was used as the solvent in all experiments. Capsanthin standard (purity 98%) was purchased from Shanghai Yuanye Bio-Technology Co., Ltd. (Shanghai, China).

### 2.2. Preparation of WPI-WalPC Pickering Emulsions with Different Blending Ratios

Whey protein isolate (WPI) and walnut protein concentrate (WalPC) were separately dissolved in ultrapure water at a concentration of 6% (*w*/*v*), stirred for 2 h and then hydrated overnight at 4 °C. WPI and WalPC were subsequently mixed at the following dry mass ratios: 0:10, 1:9, 3:7, 5:5, 7:3, 9:1 (*w*/*w*), and 10:0. Each mixture was stirred for 1 h to obtain homogeneous WPI-WalPC blended dispersions. These seven blended dispersions were then mixed with walnut oil at a constant mass ratio of 3:7 (protein dispersion:walnut oil, *w*/*w*). Each mixture was homogenized using a high-speed homogenizer (T18, IKA, Staufen im Breisgau, Germany) at 10,000 rpm for 5 min to form fresh Pickering emulsions. All homogenized samples were immediately placed in an ice bath to avoid excessive temperature rise and consequent protein denaturation. The walnut oil used in this study naturally contains endogenous antioxidants (e.g., tocopherols). However, all emulsions were prepared using the same batch and source of oil, and blank oil samples were used for baseline correction in all measurements. Therefore, these endogenous antioxidants did not interfere with the determination of capsanthin retention rate, peroxide value, or acid value.

### 2.3. Determination of Emulsion Stability

#### 2.3.1. Particle Size and ζ-Potential

Droplet size and ζ-potential were measured using a Mastersizer 2000 (Malvern, Malvern in Worcestershire, UK). The volume-weighted mean diameter D_4,3_ was calculated as described by Li et al. and Yang et al. Equation (1) [11,12]:

(1) $$D_{4,3}=\frac{\sum (n_{i}\cdot d_{i}^{4})}{\sum (n_{i}\cdot d_{i}^{3})}$$

where n_i_ denotes the number of emulsion droplets, and d_i_ represents the diameter of emulsion droplets.

#### 2.3.2. Determination of Emulsifying Activity Index (EAI) and Emulsifying Stability Index (ESI)

The EAI and ESI of the emulsions were determined following the method described by Ma et al. [13]. (The aqueous phase used for emulsion preparation was ultrapure water. No pH adjustment was performed, and no salts (e.g., NaCl) were added; therefore, the pH was the natural pH of the protein dispersion and salinity was negligible). Immediately after emulsion preparation (recorded as 0 min) and after standing for 10 min, 50 µL of emulsion was quickly withdrawn from the bottom and added to 5000 µL of 0.1% (*w*/*v*) SDS solution. The mixture was vortexed vigorously, and its absorbance at 500 nm was measured to obtain A0 and A10, respectively. A 0.1% (*w*/*v*) SDS solution was used as the blank. EAI and ESI were calculated using Equations (2) and (3):

(2) $$\mathrm{EAI}\;(m^{2}\cdot g^{-1})=\frac{2\times 2.303\times N\times A_{0}}{10^{5}\times \rho \times (1-\theta )\times L}$$

(3) $$\mathrm{ESI}\;(\min)=\frac{A_{0}}{\Delta A}\times t$$

where: $A_{0}$ is the absorbance of the emulsion immediately after dilution; N is the dilution factor (N = 101); ρ is the protein concentration (g/mL); θ is the volume fraction of the oil phase in the emulsion; L is the path length of the cuvette (L = 0.01 m); ΔA is the change in absorbance between 0 and 10 min (A_0_ – A_10_); t is the time interval (10 min).

#### 2.3.3. Dispersibility Stability Index (DSI) Measurement

The physical stability of Pickering emulsions stabilized by different WPI-WalPC ratios was assessed using a LUMisizer (TURBISCAN TOWER, Formulaction, Toulouse, France) stability analyzer via centrifugal testing, adapted from the method of Xu et al. [14]. Samples were centrifuged at 1500× *g* for 3 h at 25 °C (a condition approximately equivalent to one month of storage at room temperature). During centrifugation, transmission profiles were recorded using near-infrared light (wavelength 865 nm) at 30 s intervals. This process generated two-dimensional maps showing light transmittance as a function of sample tube position (distance from the rotation axis) and centrifugation time.

#### 2.3.4. Effect of pH on Emulsion Stability

The pH of the protein composite solutions was adjusted to 4.0, 5.0, 6.0, 7.5, and 9.0 using 1 mol/L HCl or NaOH before emulsion preparation. No additional salts were added. The ionic strength therefore varied with pH adjustment and was not independently controlled; this is acknowledged as a limitation. To determine droplet size, 1 mL of the emulsion was diluted with 9 mL of distilled water. The droplet size distribution was then measured via dynamic light scattering at a scattering angle of 90°, with an equilibration time of 120 s and a measurement temperature of 25 °C.

#### 2.3.5. Effect of Ionic Strength on Emulsion Stability

Different concentrations of NaCl (3, 60, 90, 120, and 150 mmol/L) were added to the protein composite solutions before emulsion preparation. The droplet size of the resulting emulsions was measured using the method described in Section 2.3.1.

#### 2.3.6. Effect of Temperature on Emulsion Stability

To evaluate thermal stability, Pickering emulsions were stored at different temperatures (−4 °C, 25 °C, 37 °C, 50 °C, and 60 °C) for 30 min. After storage, the samples were cooled to room temperature, and their droplet size was measured according to the procedure in Section 2.3.1.

#### 2.3.7. Effect of Storage Time on Emulsion Stability

From each of the seven emulsion samples, 25 mL of freshly prepared emulsion was placed in a small glass vial and stored at −4 °C in a refrigerator. The droplet size of the samples was measured on days 0, 7, 14, 21, and 28 following the method outlined in Section 2.3.1.

### 2.4. Preparation of Capsanthin-Loaded “Dual-Protein” Pickering Emulsion

Whey protein isolate (WPI) and walnut protein concentrate (WalPC) were individually dispersed in ultrapure water at a concentration of 6% (*w*/*v*). The dispersions were stirred for 2 h at room temperature and then hydrated overnight at 4 °C to obtain fully hydrated protein solutions. Subsequently, the two protein solutions were mixed at a mass ratio of 1:9 (WPI:WalPC) and stirred for 1 h to form a composite aqueous protein phase. Capsanthin was dissolved in walnut oil to obtain an oil phase at a concentration of 1 g/L. The aqueous and oil phases were mixed at a mass ratio of 3:7. The mixture was then homogenized using a high-speed homogenizer (T18, IKA, Staufen im Breisgau, Germany) at 10,000 rpm for 5 min in an ice bath to prepare the capsanthin-loaded Pickering emulsion. The ice bath was maintained throughout the process to minimize temperature elevation and prevent protein thermal denaturation. The capsanthin-loaded Pickering emulsion (CPE) served as the experimental group. A control group (CK) was prepared by dissolving capsanthin directly in walnut oil at the same concentration as in the experimental group (1 mg/mL).

### 2.5. Stability of Capsanthin-Loaded Pickering Emulsion

#### 2.5.1. Establishment of Capsanthin Standard Curve

A 10 mg portion of capsanthin standard was dissolved in absolute ethanol and made up to a volume of 50 mL. A 2 mL aliquot of this solution was transferred to a 25 mL volumetric flask and diluted to volume with absolute ethanol. From this secondary solution, aliquots of 0.5, 1, 2, 3, and 4 mL were separately transferred into 10 mL volumetric flasks and diluted to volume with absolute ethanol. The absorbance of these solutions was measured at 460 nm using a UV-Vis (UV-6100S, Shanghai Yuanxi Instrument Co., Ltd., Shanghai, China)spectrophotometer. The capsanthin standard curve was established: Y = 0.0119x + 0.0813 (*R*^2^ = 0.9992), where Y is absorbance and x is capsanthin concentration (µg/mL).

#### 2.5.2. Determination of Capsanthin Retention Rate

The capsanthin content was determined according to the method of Zhu et al. [15] with slight modifications. A 1.0 g sample of the Pickering emulsion or the walnut oil control (CK) was mixed with 2 mL of absolute ethanol and vortexed thoroughly. This extraction process was repeated three times until the extract became colorless. The combined extracts were transferred to a 25 mL brown volumetric flask and diluted to volume with absolute ethanol. The absorbance of the solution was measured at 460 nm, and the capsanthin content was calculated based on the standard curve. capsanthin retention rate (%) was calculated using Equation (4):

(4) $$\mathrm{Loading}\;\mathrm{Efficiency}\;(\%)\;=\frac{C_{0}}{C_{1}}$$

where: $C_{1}$ is the concentration of capsanthin in the measured sample (%); $C_{0}$ is the initial concentration of capsanthin in the emulsion sample (%).

#### 2.5.3. Photostability

To investigate the protective effect of the Pickering emulsion on capsanthin, samples from both the CPE and CK groups were placed in glass vials and exposed to light. At specified time intervals (0, 2, 4, 8, 12, 24, 48, 72 h), samples were withdrawn, and the capsanthin retention rate was determined as described in Section 2.5.2.

#### 2.5.4. Storage Stability

Samples from the CK and CPE groups were placed in glass vials and stored at 4 °C under both light-protected and light-exposed conditions. Samples were taken at days 0, 1, 3, 7, 14, and 21, and the capsanthin retention rate was determined according to the method in Section 2.5.2.

#### 2.5.5. Sterilization Stability

The prepared capsanthin-loaded Pickering emulsion and the oil control (CK) were placed in 10 mL centrifuge tubes and subjected to heat treatment and high-pressure processing (HPP). Heat treatment conditions: 65 °C for 30 min and 90 °C for 30 s. HPP conditions: 188 MPa for 14 min. After treatment, the capsanthin retention rate was determined as per Section 2.5.2.

### 2.6. Preparation of Butter Spreadable Paste with Different CPE Substitution Ratios

A capsanthin-loaded Pickering emulsion (CPE) was used to replace butter in a butter spreadable paste, employing a direct substitution method to prepare spreadable pastes with CPE substitution ratios ranging from 0% to 50%. The formulations are shown in Table 1. Before sample preparation, all reagents and equipment were sterilized. According to the formulations in Table 1, butter and CPE samples were weighed into a 250 mL beaker for each substitution ratio. The mixtures were whipped at room temperature for 5 min until uniformly blended. The resulting mixtures were then stored in an incubator at 25 °C for subsequent analysis. Figure 1 shows a schematic diagram of the overall experimental procedure in this study.

### 2.7. Quality Parameter Determination of Butter Spreadable Pastes with Different CPE Substitution Ratios

#### 2.7.1. Color Measurement

The color of the butter spreadable pastes was determined using a colorimeter (CR-400, Shenzhen Lixin Precision Instrument Equipment Co., Ltd., Shenzhen, China), following the method described by Cunha et al. [16]. The color parameters L* (lightness), a* (green-red), and b* (blue-yellow) were measured. Each sample was measured twice, and the average value was recorded. Chroma (C*) was calculated using Equation (5):

(5) $$C^{*}=\sqrt{a^{{*}^{2}}\times b^{{*}^{2}}}$$

#### 2.7.2. Determination of Centrifugal Oil Separation Rate

Approximately 1.0 g of each spread sample was accurately weighed into a 10 mL centrifuge tube. The tube was placed in a high-speed benchtop centrifuge model (TCL-20bR, Zhejiang Zhaide Instrument Equipment Co., Ltd., Hangzhou, China), brand, country and centrifuged at 8500 rpm for 15 min at 25 °C/room temperature. After centrifugation, the tube was carefully inverted and held for 5 min to allow the separated oil to drain. Any residual oil adhering to the inner wall of the tube was gently wiped off with absorbent cotton, taking care not to disturb the pellet. The mass of the separated oil was determined by weighing the tube before and after oil removal, or by collecting the drained oil. The centrifugal oil separation rate was calculated using Equation (6):

(6) $$Centrifugal\;Oil\;Separation\;Rate\;(\%)=\frac{M_{1}-M_{2}}{M_{0}}$$

where: $M_{0}$ is the initial mass of the sample (g); $M_{1}$ is the mass of the centrifuge tube with the sample before centrifugation (g); $M_{2}$ is the mass of the centrifuge tube with the sample after centrifugation and oil removal (g).

#### 2.7.3. Texture Profile Analysis (TPA)

Texture profile analysis was performed with slight modifications based on the method of Nikolova et al. [17]. A 20 g portion of the thoroughly mixed sample was placed in a 50 mL beaker. A two-cycle compression test was conducted at room temperature using a cylindrical probe at a test speed of 0.1 mm/s. Parameters such as hardness, adhesiveness, cohesiveness, springiness, and gumminess were derived from the resulting force-time curve (TMS-PILOT, USA; distributed by Beijing Yingsheng Hengtai Technology Co., Ltd., Beijing, China. The detailed operating conditions were as follows: probe 75 mm disc extrusion probe, pre-test speed 60 mm/min, test speed 60 mm/min, post-test speed 10 mm/s, compression 40%, trigger force 0.75 N, interval between two compressions 5 s, and trigger type automatic).

#### 2.7.4. Peroxide Value (PV) and Acid Value (AV) Determination

The acid value (AV) and peroxide value (POV) of the samples were determined according to the methods described by Chen et al. [18].

#### 2.7.5. Sensory Evaluation 

Sensory evaluation was conducted based on industry standards for spreadable pastes. A total of ten trained panelists with backgrounds in food science were recruited. Prior to the formal evaluation, panelists were trained according to standard sensory protocols, including attribute recognition, intensity scaling, and the use of the 9-point hedonic scale. The evaluation covered five attributes: color, aroma, taste, spreadability, and overall acceptability. Samples were presented in randomized order and labeled with random three-digit codes. Panelists evaluated the samples independently in individual booths under white light at room temperature (25 ± 1 °C), and rinsed with water between samples. Each attribute was scored using a 9-point hedonic scale (1 = dislike extremely, 5 = neither like nor dislike, 9 = like extremely). The sensory score for each attribute was expressed as the mean of the scores given by all panelists.

### 2.8. Data Analysis

All measurements were performed in triplicate (*n* = 3), and all results were presented as mean ± standard deviation. Prior to statistical analysis, the normality of data was assessed using the Shapiro–Wilk test, and the homogeneity of variance was evaluated using Levene’s test. Statistical analysis was performed using one-way analysis of variance (ANOVA) followed by Duncan’s multiple range test for post hoc comparisons, with IBM SPSS Statistics 27.0 software. Data processing and analysis were conducted using SPSS 27.0 and Origin 2025. Statistical significance was defined as *p* < 0.05. Different lowercase letters in the figures and tables indicate significant differences among groups (*p* < 0.05).

## 3. Results

### 3.1. Emulsion Stability Analysis

#### 3.1.1. Interfacial Behavior Analysis

Figure 2 shows the Emulsifying Activity Index (EAI) and Emulsifying Stability Index (ESI) of Pickering emulsions prepared with different ratios of WPI to WalPC (Walnut protein concentrate, WalPC). The EAI reflects the adsorption capacity of solid emulsifiers at the oil–water interface, while the ESI indicates their ability to maintain emulsion stability over time [13]. As shown in Figure 2, the EAI and ESI of Pickering emulsions prepared with different WPI-WalPC ratios differed significantly (*p* < 0.05). The emulsion prepared with a WPI to WalPC ratio of 1:9 exhibited the highest EAI (48.55 m^2^/g) and ESI (240.91 min), whereas the ratio of 7:3 resulted in the lowest values (EAI: 8.46 m^2^/g; ESI: 18.97 min). Compared to emulsions stabilized solely by WalPC or WPI, the 1:9 ratio increased EAI by 51.01% and 68.34%, and ESI by 141.13% and 160.44%, respectively. These results indicate that the 1:9 ratio yields the optimal emulsifying activity and stability. This enhancement can be attributed to a synergistic interfacial interaction between the two proteins. The minor component, WPI, with its high solubility and rapid adsorption kinetics, preferentially adsorbs at the oil–water interface, forming a dense primary film that increases the mechanical strength of the interfacial layer. The dominant component, WalPC, with its flexible molecular structure, embeds into this interfacial film and extends into the aqueous phase, providing significant steric hindrance [19]. At the optimal ratio (1:9, WPI-WalPC), this synergistic effect co-constructs a stable interfacial architecture combining both strength and thickness. The emulsifying performance at this optimal ratio surpasses that reported for similar (Soy Protein Isolate, SPI) SPI:WPI systems in the literature [20]. Conversely, the poorest performance at the 7:3 ratio may be due to excessive competitive adsorption by WPI, leading to the formation of a less stable interfacial structure [20]. These findings demonstrate that not all protein combinations result in positive synergistic effects.

#### 3.1.2. Physical Stability of Emulsions

The physical stability of Pickering emulsions prepared with different WPI:WalPC ratios was evaluated using a LUMiSizer stability analyzer. Figure 3 illustrates the evolution of the transmission profiles, in which the color gradient from red to green visually represents changes in sample light transmission over time. All emulsions initially exhibited low transmission, indicating a uniform system. As centrifugation progressed, component migration accelerated in less stable systems, leading to alterations in light transmission. As shown in Figure 3, the Pickering emulsion prepared with a WP:WalPC ratio of 1:9 demonstrated excellent centrifugal stability. Its transmission profile showed minimal change throughout the process, maintaining consistently low transmission across the observation range. This result is highly consistent with the conclusions drawn from the instability index analysis and corroborates the previously reported characteristics of the smallest droplet size (Our previous data showed that the droplet sizes of WPI–WalPC emulsions at different ratios ranged from 253.03 to 1024.33 nm, with the smallest droplet size (253.03 nm) observed at a WPI:WalPC ratio of 1:9) and the highest modulus from rheological measurements [21]. In contrast, emulsions with other ratios exhibited a significant increase in transmission in the middle region during centrifugation, which could be ascribed to intensive droplet migration and creaming. According to the principle of LUMiSizer analysis, faster and more pronounced changes in transmission indicate poorer physical stability of the system [14]. In summary, the Pickering emulsion prepared with a WPI-WalPC ratio of 1:9 exhibited optimal performance in terms of droplet size distribution, rheological properties, and accelerated centrifugal stability testing. Therefore, this optimal ratio system was selected as the carrier for subsequent capsanthin loading.

#### 3.1.3. Influence of Various Factors on Emulsion Stability

To evaluate the stability of the WPI-WalPC composite Pickering emulsions, the effects of pH, ionic strength, temperature, and storage time on their volume-weighted mean droplet diameter (D_4,3_) were systematically investigated. As shown in Figure 4a, pH had a significant (*p* < 0.05) impact on emulsion droplet size. Overall, emulsions under acidic conditions (pH 4.0, 5.0, 6.0) exhibited smaller droplet sizes, while the sizes increased under neutral and alkaline conditions. This is likely attributed to reduced electrostatic stabilization, leading to droplet aggregation or flocculation [22,23]. Among all systems, the emulsion with a WPI-WalPC ratio of 1:9 demonstrated the best pH stability, showing the smallest variation range in D_4,3_ (363.22–624.92 nm). In contrast, single-protein emulsions stabilized by WalPC (491.50–984.43 nm) or WPI (506.16–983.85 nm) exhibited much broader ranges of size variation.

Salt ions can reduce the surface potential of particles through electrostatic screening, thereby weakening electrostatic repulsion between droplets and inducing flocculation or aggregation [24]. As shown in Figure 4b, the droplet sizes of all emulsions changed significantly across the NaCl concentration range of 3~150 mmol/L. Emulsions stabilized by single proteins were most sensitive to ionic strength. The droplet size of the WalPC-stabilized emulsion increased substantially from 1024.11 nm to 1884.60 nm, while that of the WPI-stabilized emulsion increased from 1124.01 nm to 1566.18 nm. This is primarily because NaCl addition weakens the electrostatic attraction between oppositely charged groups within the WPI and WalPC molecules, loosening the composite interfacial structure. More importantly, the electrostatic screening effect directly reduces the electrostatic repulsion between droplets, thereby exacerbating aggregation [25]. Studies by Wu et al. [26] also indicated limitations of single proteins in stabilizing interfaces, making emulsions prone to destabilization under increased ionic strength. In contrast, the emulsion prepared with a WPI-WalPC ratio of 1:9 exhibited the best salt stability, with the smallest variation in droplet size. This suggests that the interfacial structure formed at this specific ratio is more compact and effective in resisting salt-induced destabilization.

Temperature is a key factor affecting emulsion stability, primarily by altering protein conformation and interfacial behavior [27]. As shown in Figure 4c, this study systematically evaluated the effects of low (−4 °C), ambient (25 °C, 37 °C), and high temperatures (50 °C, 60 °C) on emulsion stability. All emulsions maintained relatively stable droplet sizes within the temperature range of −4 °C to 25 °C, suggesting good structural integrity and emulsifying stability of the WPI-WalPC composite proteins within this interval. However, droplet sizes increased significantly at 50 °C and 60 °C. This is likely due to protein denaturation at high temperatures, which disrupts the compact structure of the interfacial layer, leading to emulsion aggregation and destabilization [27]. Notably, among the different blending ratios, the emulsion with a WPI-WalPC ratio of 1:9 exhibited the best thermal stability. It maintained the most stable droplets at −4 °C., but also showed a significantly smaller increase in droplet size under the stress of 50 °C and 60 °C compared to systems with other protein ratios. This indicates that this specific blending ratio forms an interfacial film that is more thermodynamically stable and mechanically robust, thus more effectively resisting heat-induced destabilization.

Long-term storage stability is crucial for assessing the practical application potential of Pickering emulsions. As shown in Figure 4d, the droplet sizes of all emulsions exhibited an increasing trend over a 28-day storage period, but the stability varied significantly among systems with different blending ratios. The emulsion with a WPI-WalPC ratio of 1:9 performed optimally, showing a significantly smaller increase in droplet size compared to other ratios, demonstrating excellent long-term stability. In contrast, the emulsion stabilized solely by WPI exhibited the poorest stability, with its droplet size increasing from 1137.29 nm to 1377.78 nm, representing the largest increase. This significant difference in stability primarily stems from the synergistic interaction between WPI and WalPC at the 1:9 ratio, which forms a more compact and robust interfacial structure. This structure more effectively hinders Ostwald ripening and suppresses droplet aggregation, thereby demonstrating the significant advantage of the protein blending strategy in enhancing the long-term storage stability of Pickering emulsions [28].

### 3.2. Stability Analysis of Capsanthin-Loaded Pickering Emulsion

#### 3.2.1. Photostability Analysis

Capsanthin is highly susceptible to photodegradation. Constructing a delivery system can reduce its direct exposure to light, thereby enhancing its photostability [29]. To verify the protective effect of the Pickering emulsion, the loading efficiency of capsanthin in the Pickering emulsion (CPE) under light exposure (0~72 h) was measured. A control (CK) was prepared by dispersing capsanthin directly in walnut oil. As shown in Figure 5, the capsanthin retention rate in both groups decreased with prolonged light exposure, whereas the CPE group demonstrated significantly better stability than the CK group. After 72 h of light exposure, the loading efficiency in the CK group dropped sharply from 88.58% to 45.45%. In contrast, the loading efficiency in the CPE group remained at 81.5% (initial value: 93.52%). This indicates that the CPE system provides significant photoprotection for capsanthin. This favorable effect is attributed to the dense, solid particle interfacial layer formed by the WPI-WalPC composite protein particles at the oil–water interface. This layer effectively scatters and blocks light, providing a physical barrier for the encapsulated capsanthin and thereby substantially slowing the photodegradation rate.

#### 3.2.2. Storage Stability Analysis

Figure 6a illustrates the change in capsanthin retention rate over time for the CK group and the CPE group under storage at 4 °C in the dark. The results indicated that although the loading efficiency decreased over time for both groups, the stability of the CPE group was significantly superior to that of the CK group. At the initial time point (0 d), the loading efficiencies of the CK and CPE groups were 88.58% and 93.52%, respectively. After 30 days of storage, the loading efficiency of the CK group decreased to 60.25% (a cumulative decrease of 28.33%), whereas the CPE group still maintained a loading efficiency of 78.22% (a cumulative decrease of 15.30%). The difference between the two groups was significant (*p* < 0.05).

Figure 6b shows the change in capsanthin retention rate over time for the CK and CPE groups under storage at 4 °C with light exposure. Under this condition, the decline in loading efficiency was more pronounced for both groups; however, the stability of the CPE group remained significantly better than that of the CK group. After 30 days of storage, the loading efficiency of the CK group decreased to 50.25% (a cumulative decrease of 38.33%), while that of the CPE group remained at 70.66% (a cumulative decrease of 22.86%). The difference between the two groups was significant (*p* < 0.05). Comparing the two storage conditions, the decrease in loading efficiency for the CPE group was more significant under light exposure than in the dark. This further confirms that light exposure is a key environmental factor accelerating capsanthin degradation and thus reducing loading efficiency. This is attributed to the sensitivity of the conjugated double bonds in capsanthin molecules to light energy, which makes them prone to isomerization and oxidative degradation under illumination, leading to desorption from the carrier or structural breakdown of the pigment itself [29].

These findings further confirm that the Pickering emulsion prepared with the optimal WPI-WalPC dual-protein ratio (1:9) provides significant protection for capsanthin. This emulsion system forms a dense and irreversibly adsorbed protein particle barrier at the oil–water interface. This barrier not only effectively blocks the penetration of external degradation factors such as light and oxygen, but also inhibits the diffusion and desorption of capsanthin molecules from the interface into the continuous phase [30]. Accordingly, it significantly slows the degradation rate of the pigment and reduces the loss of its loading efficiency during storage.

Figure 6c systematically showed the effects of three sterilization methods—pasteurization (60 °C/30 min), high-temperature short-time (HTST) treatment (90 °C/30 s), and high-pressure processing (HPP) on the capsanthin retention rate in both the CPE and CK groups. This evaluation aimed to identify a suitable sterilization method for potential industrial production.

The impact of the three sterilization methods on capsanthin retention was significantly different (*p* < 0.05). Following all three treatments, the capsanthin retention rate in the CPE group remained consistently higher than in the CK group, further corroborating the protective effect of the Pickering emulsion system. Specifically, the capsanthin retention rates for the CK group after 60 °C/30 min, 90 °C/30 s, and HPP treatments were 78.10%, 75.54%, and 81.34%, respectively. In contrast, the corresponding retention rates for the CPE group were 86.15%, 86.05%, and 90.20%, which were significantly higher than those of the CK group (representing increases of 10.31%, 13.94%, and 10.89%, respectively). This improvement is primarily attributed to the dense particle barrier formed by the WPI-WalPC dual-protein composite particles at the oil–water interface, which reduces the contact between the pigment and the external environment during sterilization, thereby minimizing degradation losses [31].

Notably, increasing the thermal sterilization temperature clearly undermined pigment stability. After 90 °C/30 s HTST, both groups showed lower capsanthin retention than after 60 °C/30 min, and the drop was much steeper in CK. This is because the conjugated double bonds of capsanthin are heat-sensitive, and high temperature accelerates thermal-oxidative degradation [29]. CPE, however, declined more moderately, owing to the interfacial film that acts as a physical barrier and slows heat-induced oxidation. In contrast, after HPP treatment, both CK and CPE retained more capsanthin than after either thermal treatment. CPE reached 90.20% retention. HPP mainly disrupts non-covalent bonds—hydrogen bonds and hydrophobic interactions—with little effect on the covalent structure of small molecules like capsanthin, so color and chemical integrity are largely preserved [32]. Additionally, the high-pressure environment promotes tighter packing of WPI–WalPC composite particles at the interface, which strengthens the emulsion network and indirectly improves pigment retention [33].

### 3.3. Quality Evaluation of Spreadable Butter Pastes with Different CPE Substitution Ratios

#### 3.3.1. Color Analysis

To quantitatively characterize the color changes in spreadable pastes with different CPE substitution ratios, the lightness (L*), red-green value (a*), yellow-blue value (b*), and chroma (C*) of the samples were measured using a colorimeter. As shown in Table 2, the lightness value (L*) systematically and significantly decreased as the substitution ratio increased from 0% to 50%, whereas notable differences were observed among the samples with different substitution ratios. This decrease is attributed to the fact that the control group (0% substitution) primarily consists of a crystalline fat network and a small number of air bubbles, which strongly scatter visible light, resulting in a bright, milky white appearance [34]. The introduction of CPE partially replaces butter with an oil-in-water emulsion stabilized by WPI-WalPC. The WPI-WalPC particles themselves exhibit a pale yellow color, and the emulsion droplets they form alter light absorption and scattering efficiency, leading to an overall reduction in lightness [35].

Concurrently, the redness value (a*) increased significantly with increasing substitution ratio, which is attributed to the increased content of capsanthin. Furthermore, the chroma (C*) also increased accordingly, indicating that the addition of CPE significantly enhanced the color vividness of the product, achieving a visual representation of the functional component. The appearance photographs of the spreads with different CPE substitution ratios are shown in Figure 7, from which the above results can be directly observed.

**Figure 7:**
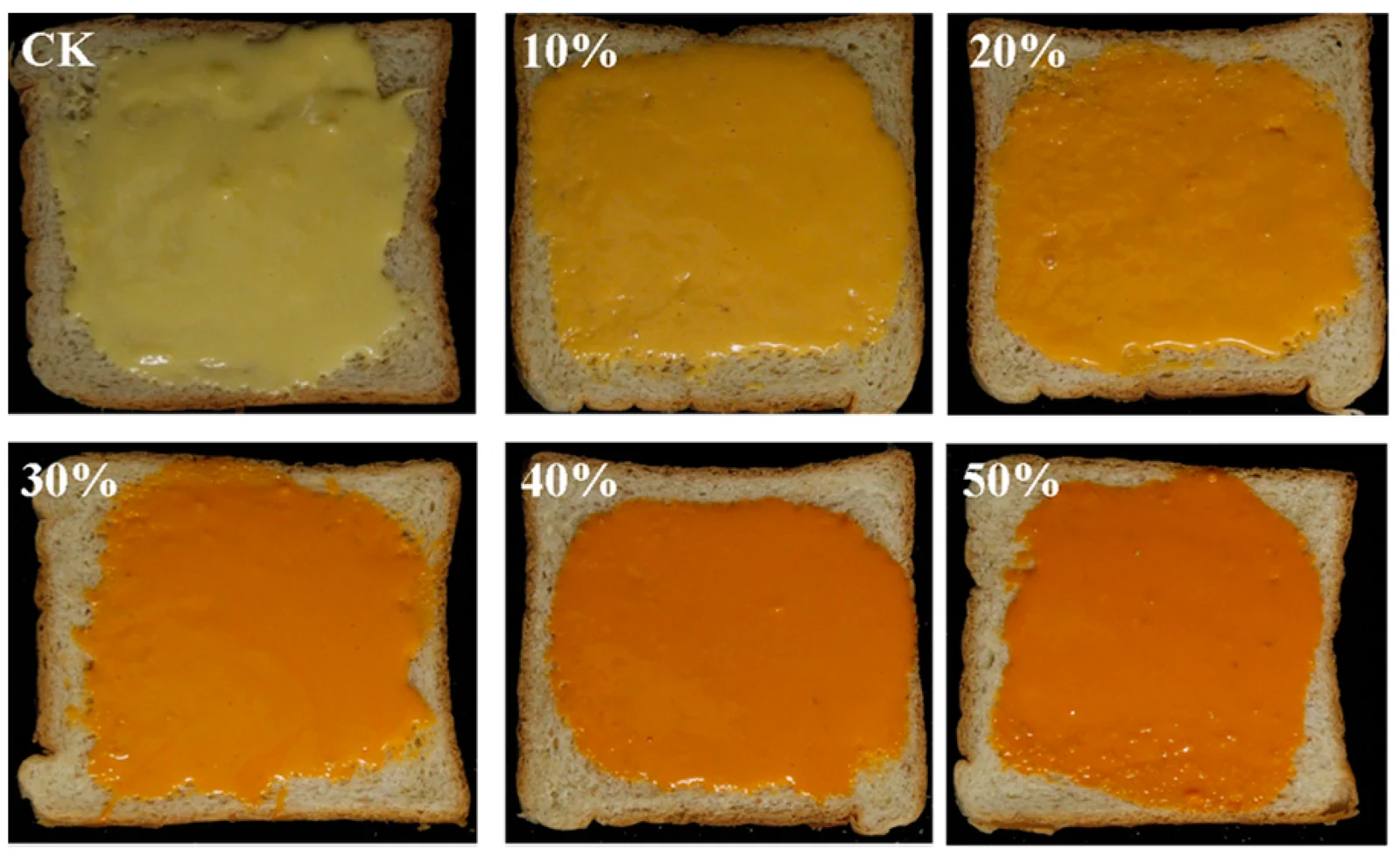
Comprehensive quality attributes of butter spreads with different CPE substitution ratios: spreadability on bread surface. (CPE substitution ratios were 0%, 10%, 20%, 30%, 40%, and 50%).

#### 3.3.2. Analysis of Centrifugal Oil Separation Rate

The centrifugal oil separation rate is a critical indicator for assessing the stability of food emulsions. A lower separation rate signifies a more stable system. As shown in Figure 8a, the centrifugal oil separation rate of the samples exhibited a significant decreasing trend as the CPE substitution ratio increased from 0% to 50%. The control group (0% substitution, pure butter) showed the highest separation rate (99.67%). With increasing CPE addition, the centrifugal oil separation rate decreased significantly (0.2~0.5%), indicating markedly enhanced emulsion stability. This improvement is attributed to the three-dimensional gel network and the interfacial protein layer formed by the CPE system, which effectively entraps water and oil, strengthens the internal structural integrity of the system, and improves its resistance to centrifugal separation, thereby enhancing physical stability [36]. The results demonstrate that the incorporation of CPE not only did not compromise system stability but also significantly improved the mechanical stability and storage quality of the spreadable paste by constructing a reinforced network structure.

#### 3.3.3. Texture Analysis

The texture characteristics of a spreadable paste, such as hardness and chewiness, are central to determining its sensory quality and consumer acceptability. As shown in Table 3, the texture properties of spreadable pastes prepared with CPE substituting for butter differed significantly (*p* < 0.05) from the full-butter control (0% substitution). The full-butter sample exhibited the highest hardness (0.35 N), which is likely due to its dense internal fat crystal network. This finding aligns with Ziarno et al. [37], who reported that a high solid fat content increases hardness and reduces spreadability in butter spreads.

As the CPE substitution ratio increased, the hardness of the samples decreased from 0.35 N to 0.12 N, while spreadability correspondingly improved. This is attributed to the oil-in-water gel network formed by CPE, which is more flexible and deformable than the continuous fat phase, thereby reducing the overall firmness of the product. This improvement makes the product easier to spread directly from refrigeration, addressing a key practical drawback of traditional butter.

Notably, despite the decrease in hardness, the chewiness of the 30% substitution sample (1.10 mJ) was not significantly different from that of the control (1.08 mJ). This indicates that the energy required for oral processing and the perceived oral fullness were largely preserved, and the product did not become crumbly or hollow. Therefore, the 30% substitution sample achieved a balance between reduced hardness and maintained oral satisfaction, rather than simply retaining a “dense texture.” This observation is consistent with Yılmaz and Öğütcü et al. [38], who emphasized the close relationship between hardness and spreadability of fat-based products, and the importance of precise texture modulation in maintaining desirable mouthfeel. A limitation of this study is that sensory hardness and spreadability were not separately quantified; future work should correlate TPA parameters with sensory texture attributes to further validate these findings.

#### 3.3.4. Acid Value (AV) and Peroxide Value (POV)

AV and POV are key indicators for assessing the degree of lipid oxidation. Higher values indicate more severe lipid rancidity [39]. As shown in Figure 8b, as the CPE substitution ratio increased from 10% to 30%, both the AV and POV of the samples decreased continuously, reaching their lowest values at a 30% substitution ratio (AV = 0.318 mg KOH/g fat, POV = 0.078 g/100 g). Compared to the control group, these values represented reductions of 65% and 79%, respectively. This significant improvement is primarily attributed to the dense three-dimensional network structure formed by the Pickering emulsion system, which physically impedes contact between oxygen and lipids, thereby effectively delaying lipid oxidation [40]. When the substitution ratio exceeded 30%, a slight increase in AV and POV was observed. This is likely because an excess of dual-protein particles failed to be completely adsorbed at the interface, leading to aggregation within the aqueous or oil phase. This phenomenon could weaken the uniformity and barrier properties of the interfacial film. Furthermore, it might cause localized oil droplet coalescence and separation, exposing the lipids more directly to air and making them more susceptible to oxidation [41]. Despite this slight increase at higher substitution levels, the AV and POV values for all substitution groups remained significantly lower than those of the control group. Importantly, all values were well within the permissible limits of the national food safety standards (e.g., AV ≤ 3.0 mg KOH/g fat, POV ≤ 0.25 g/100 g). This trend is consistent with the findings of Chen et al. [42], who reported that substituting traditional fat with protein-based Pickering emulsions effectively inhibited lipid oxidation. In conclusion, the addition of the dual-protein composite particles significantly enhanced the oxidative stability of the system. The 30% substitution ratio demonstrated the optimal inhibitory effect. Although higher substitution ratios showed a slight decline in efficacy compared to the 30% optimum, they still performed better than the control group. These results indicate that this system possesses good application potential for delaying the oxidative deterioration of lipids.

#### 3.3.5. Sensory Evaluation Analysis

Figure 8c presents the sensory scores for the six spreadable paste formulations. A comprehensive evaluation was conducted based on five attributes: color, aroma, taste, spreadability, and overall acceptability. The results indicate significant differences in sensory characteristics among the samples, with none exhibiting off-flavors. Specifically, the 50% substitution group achieved the highest score for color. However, its aroma score showed a more noticeable decline, and its overall acceptability was lower than that of the 30% and 40% substitution groups. This suggests that while a higher substitution ratio is beneficial for enhancing product color, it may also negatively impact flavor, thereby reducing overall acceptability. In contrast, the 30% substitution group performed outstandingly in terms of overall acceptability, taste, and spreadability. Its scores were significantly higher than those of groups with higher substitution ratios (e.g., 50%) and, compared to the control group, it effectively reduced greasiness while better retaining the characteristic buttery aroma. This formulation exhibited favorable sensory quality and functional attributes, achieving the highest and most balanced overall acceptability. In practical production, the rational use of food additives could further enhance the color and flavor of the product. In summary, the sample with a 30% substitution ratio demonstrated the most advantageous overall sensory profile, offering consumers a more pleasurable eating experience and showing greater potential for development.

## 4. Discussion

The superior performance of the 1:9 WPI:WalPC ratio can be largely attributed to the complementary characteristics of animal and plant proteins. WPI is a globular protein with high interfacial activity and rapid adsorption kinetics, whereas WalPC is rich in hydrophobic residues and disulfide bonds, which contribute to structural rigidity and network strength. At the 1:9 ratio, the two proteins act synergistically and assemble at the oil–water interface, forming a dense and viscoelastic film that combines the rapid adsorption of WPI with the structural reinforcement of WalPC [19]. Gao et al. investigated a similar SPI–WPI system and reported an ESI of 20 min. In contrast, the WPI–WalPC (1:9) system in this study achieved an ESI of 240.91 min, indicating superior emulsifying performance [20].Notably, our system achieves optimal stabilization through simple physical blending, without additional cross-linking agents. When applied as a butter substitute in spreadable pastes, the optimized Pickering emulsion effectively reduced the dependence on saturated fats while maintaining desirable texture, spreadability, and sensory acceptability. This advantage is particularly relevant because conventional fat replacers, such as oleogels and protein–polysaccharide complexes, often suffer from poor oxidative stability or off-flavors. For instance, O’Sullivan et al. [43] noted that oleogels can be prone to lipid oxidation, leading to rancidity and undesirable sensory attributes. Warnakulasuriya and Nickerson et al. [44] reviewed plant protein–polysaccharide complexes and highlighted that off-flavors from plant proteins remain a major challenge for their application in fat-reduced foods. Similarly, Roland et al. [45] reported that pulse proteins often carry undesirable beany or bitter notes that can negatively affect consumer acceptance. In contrast, the WPI–WalPC Pickering emulsion developed in this study forms a dense interfacial film and a three-dimensional network that effectively retard lipid oxidation and mask off-flavors, thereby providing a cleaner sensory profile than conventional fat replacers. Our emulsion provided superior protection for capsanthin, as evidenced by higher retention under light, storage, and thermal sterilization treatments compared to the oil control. This protection is attributed to the interfacial film and three-dimensional network that act as physical barriers against oxygen, light, and free radicals.

Nevertheless, several limitations of this study should be acknowledged. First, the partition coefficient of capsanthin was not determined. Second, direct microstructural evidence was not obtained to confirm whether the 30% substitution sample forms a W/O/W multiple emulsion or undergoes a partial phase inversion; therefore, the O/W-to-W/O transition was not experimentally verified. Third, other spread formulations based on different fat replacers or protein systems were not compared. Fourth, long-term storage stability beyond 21 days was not evaluated. Future work should address these gaps, particularly the interfacial stabilization mechanism using CLSM/cryo-SEM and contact angle measurements, the microstructural evolution of the continuous phase, and the scalability of the emulsion for industrial spread production.

## 5. Conclusions

In summary, this study successfully developed a dual-protein Pickering emulsion stabilized by WPI-WalPC blends, and the optimal WPI-WalPC ratio was determined to be 1:9 based on its superior functional properties and stability. The results revealed a close relationship between emulsion stability and functional performance, where the enhanced interfacial stability provided by the optimized protein ratio served as the structural basis for improved bioactive protection and controlled lipid release. The formation of a robust protein particle-stabilized interface effectively prevented droplet aggregation and phase separation, thereby improving the functional properties of the emulsion system. The optimized Pickering emulsion was further used as a carrier for capsanthin. Compared with capsanthin directly incorporated into walnut oil, the Pickering emulsion exhibited enhanced capsanthin retention under different environmental stresses, demonstrating its superior protective capacity for lipophilic pigments. Finally, the capsanthin-loaded Pickering emulsion was successfully applied as a butter substitute in spreadable paste formulations. The incorporation of the emulsion improved spreadability, sensory properties, oxidative stability, and functional characteristics while reducing the dependence on conventional saturated fats. Overall, this study demonstrates that optimizing protein composition is an effective strategy for constructing multifunctional Pickering emulsions, in which enhanced structural stability acts as the foundation for improved delivery performance and food application potential.

## Figures and Tables

**Figure 1:**
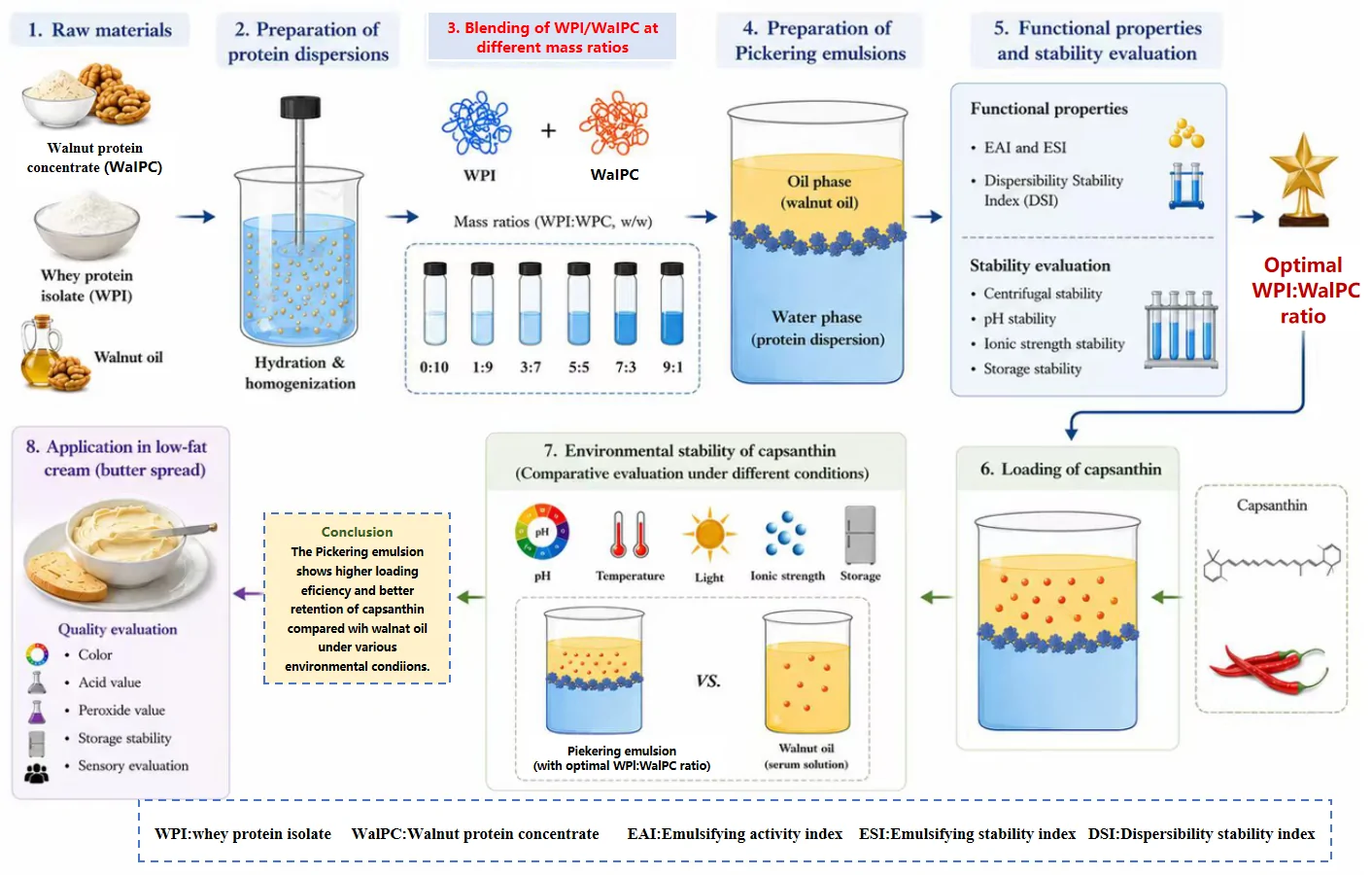
Schematic diagram of the experimental procedure, including preparation of WPI–WalPC blended dispersions, formation of capsanthin-loaded Pickering emulsions, characterization of emulsion properties, and application in butter spreads as a fat substitute.

**Figure 2:**
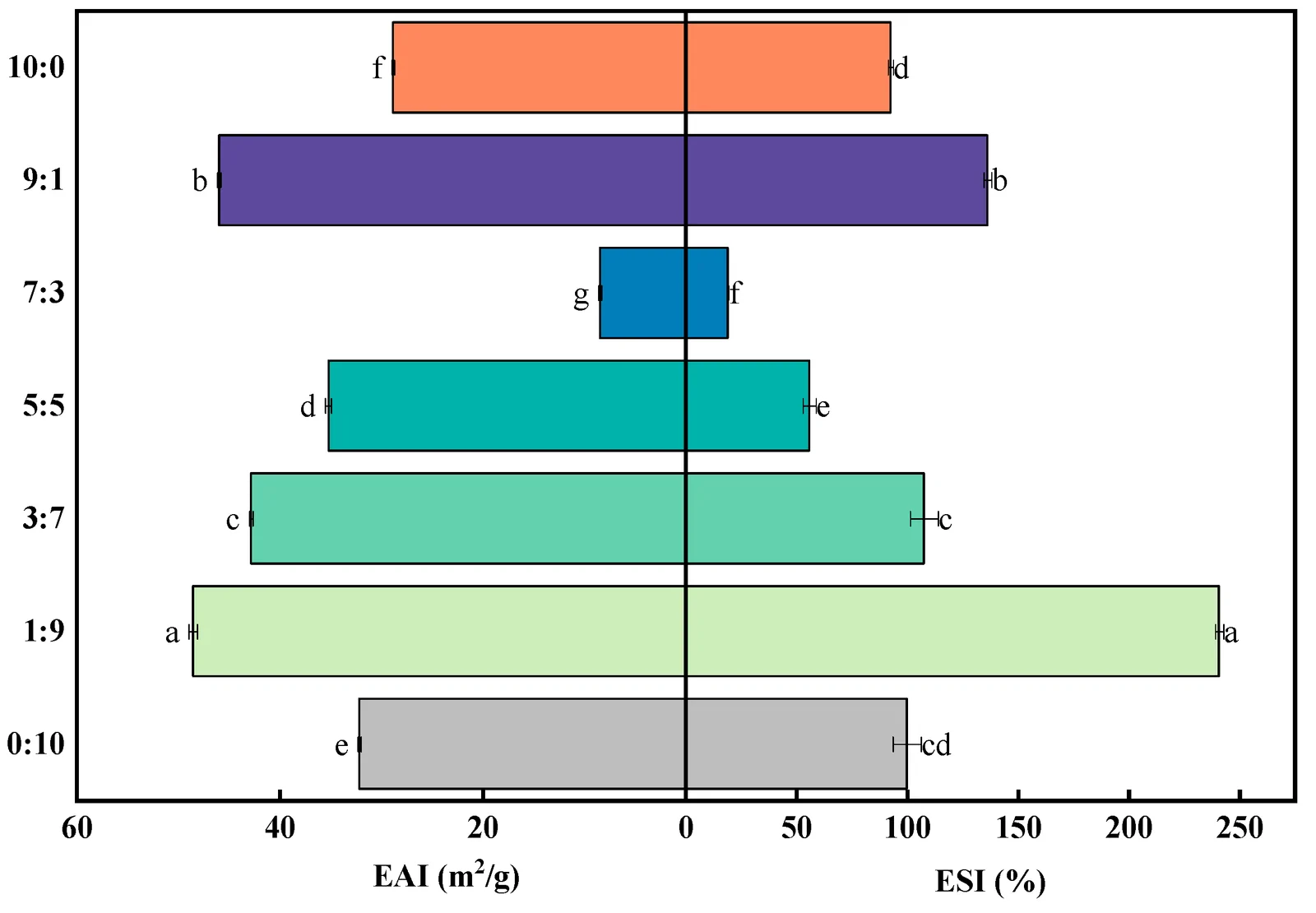
Emulsifying activity index (EAI) and emulsion stability index (ESI) of Pickering emulsions prepared with different WPI:WalPC ratios (0:10, 1:9, 3:7, 5:5, 7:3, 9:1, and 10:0). Data are presented as mean ± standard deviation (*n* = 3). Different lowercase letters indicate significant differences among ratios (*p* < 0.05). WPI, whey protein isolate; WalPC, walnut protein concentrate.

**Figure 3:**
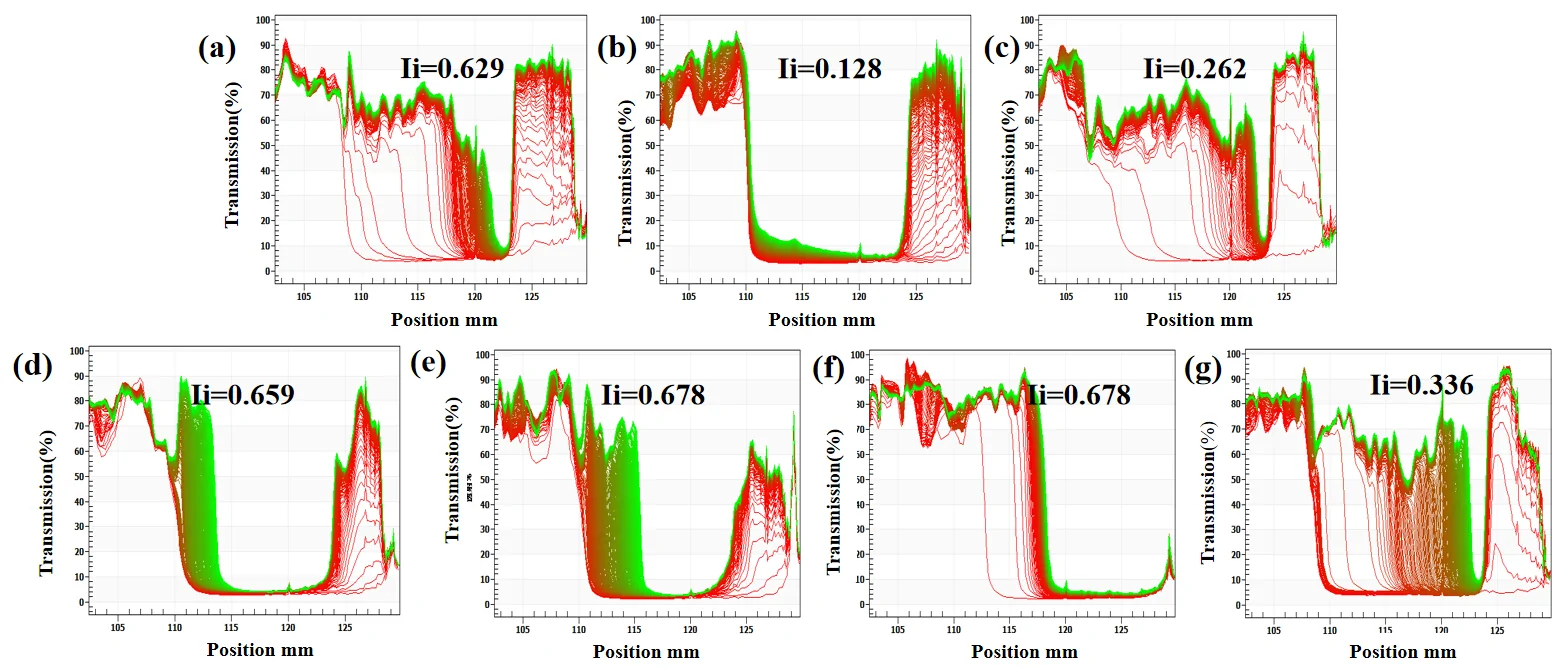
Light transmittance cumulative curves and instability index (Ii) of Pickering emulsions prepared with different WPI-WalPC ratios. (**a**–**g**) denote WPI-WalPC ratios of 0:10, 1:9, 3:7, 5:5, 7:3, 9:1, and 10:0, respectively. (Transmittance curves were recorded over 180 min; Ii values are shown as an inset or bar plot; Red curves represent the first scan at the beginning of centrifugation; green curves represent the last scan at the end of centrifugation. Intermediate colors represent successive scans during centrifugation. Data are mean ± SD (*n* = 3).

**Figure 4:**
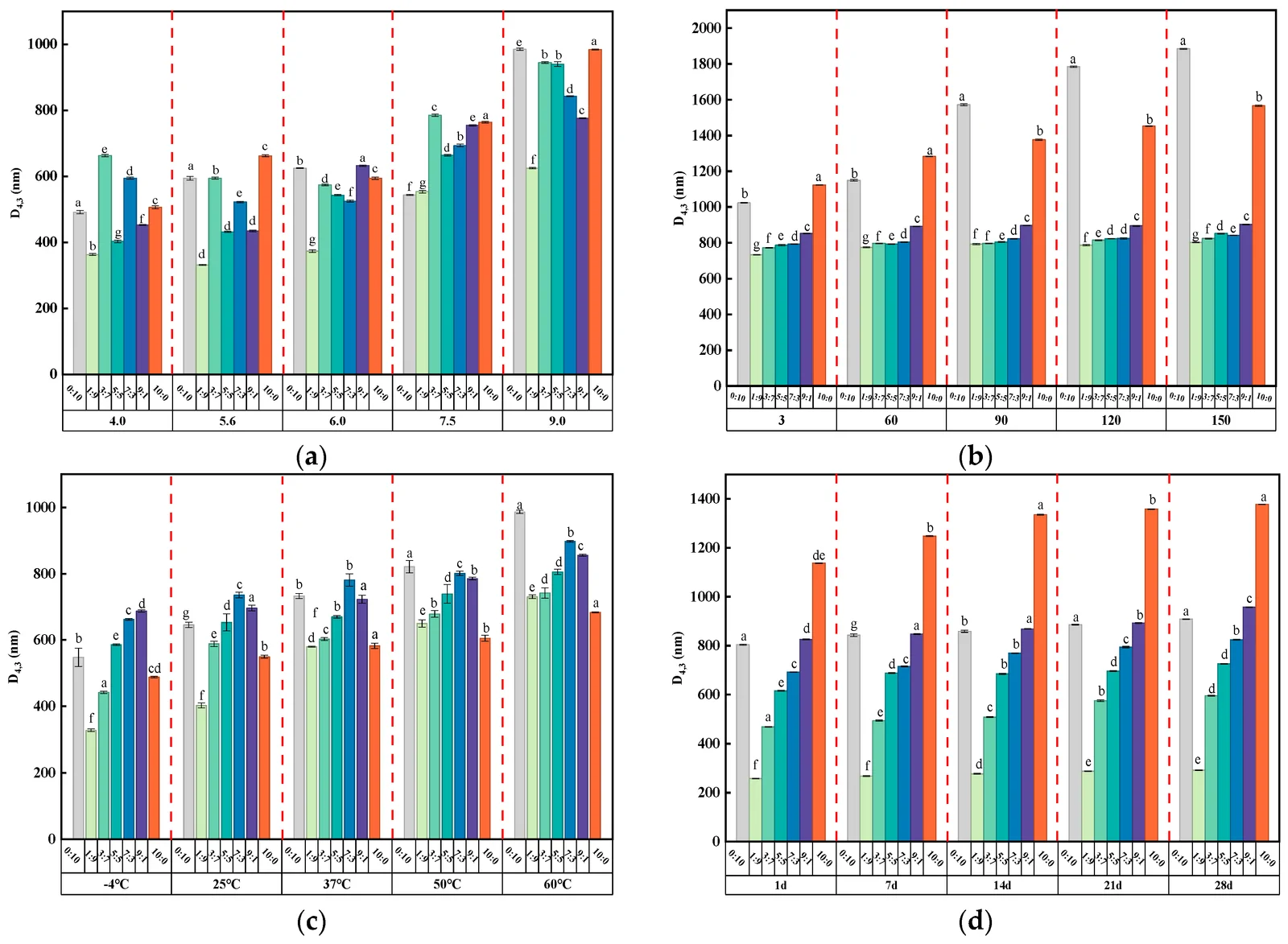
Effects of pH, salt ion concentration, temperature, and storage time on the droplet size of Pickering emulsions stabilized with different WPI:WalPC ratios. (**a**) pH range 4.0–9.0; (**b**) NaCl concentration 30–150 mM; (**c**) temperature −4–60 °C; (**d**) storage time 1–28 days. Data are presented as mean ± standard deviation (*n* = 3). Different lowercase letters indicate significant differences among groups (*p* < 0.05).

**Figure 5:**
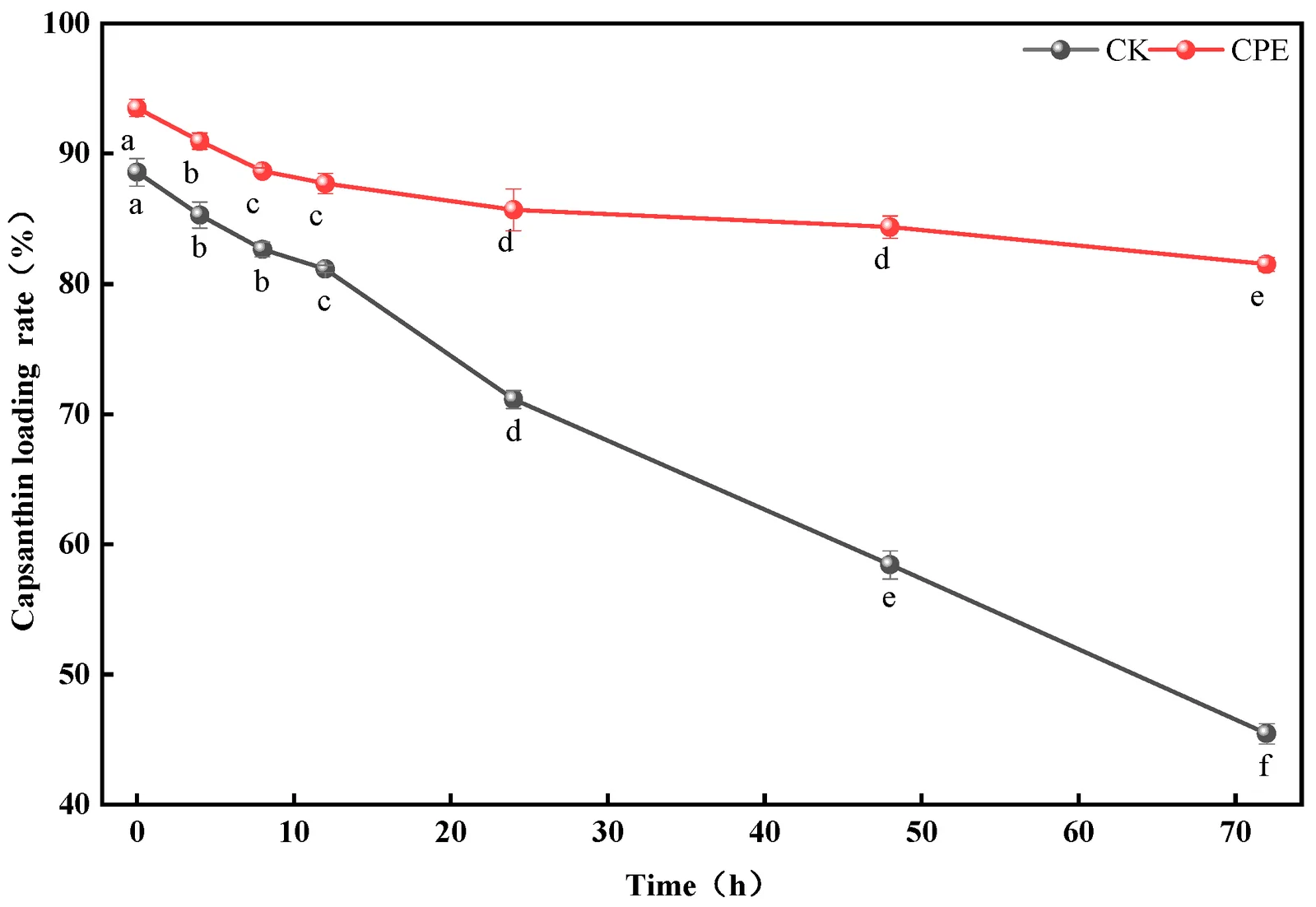
Evaluation of capsanthin-loaded Pickering emulsions: loading efficiency under light exposure. The capsanthin-loaded Pickering emulsion (CPE) and oil control (CK) were exposed to light for 0, 2, 4, 8, 12, 24, 48, and 72 h. Data are presented as mean ± standard deviation (*n* = 3). Different lowercase letters indicate significant differences between CPE and CK at the same time point (*p* < 0.05). CPE, capsanthin-loaded Pickering emulsion; CK, oil control.

**Figure 6:**
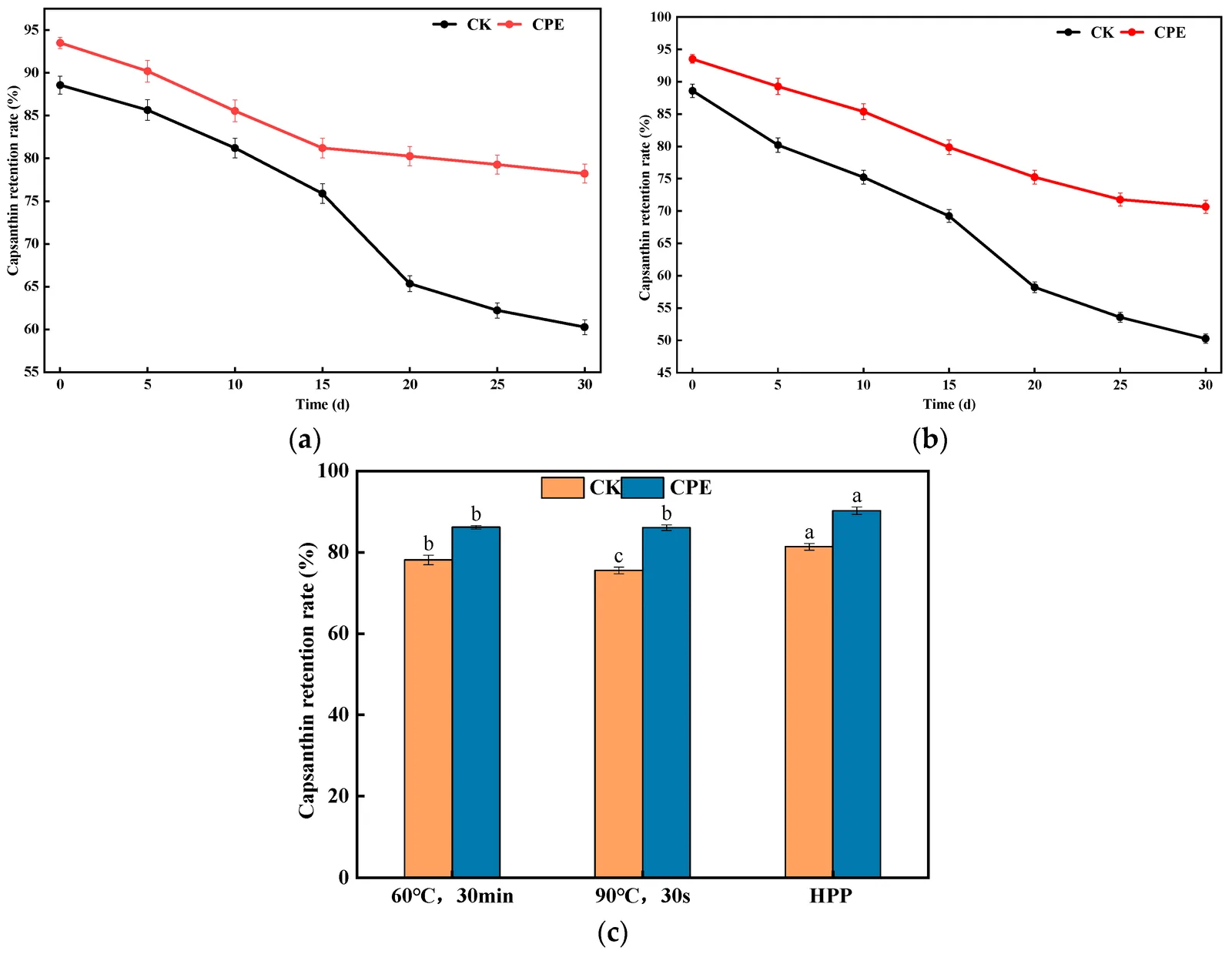
Evaluation of capsanthin-loaded Pickering emulsions: (**a**) loading efficiency under light exposure; (**b**) loading efficiency under different storage conditions (4 °C, dark; 4 °C, light); (**c**) loading efficiency under different thermal sterilization conditions (65 °C/30 min; 90 °C/30 s; 188 MPa/14 min). The capsanthin-loaded Pickering emulsion (CPE) and oil control (CK) were compared. Data are presented as mean ± standard deviation (*n* = 3). Different lowercase letters indicate significant differences among treatments (*p* < 0.05). CPE, capsanthin-loaded Pickering emulsion; CK, oil control.

**Figure 8:**
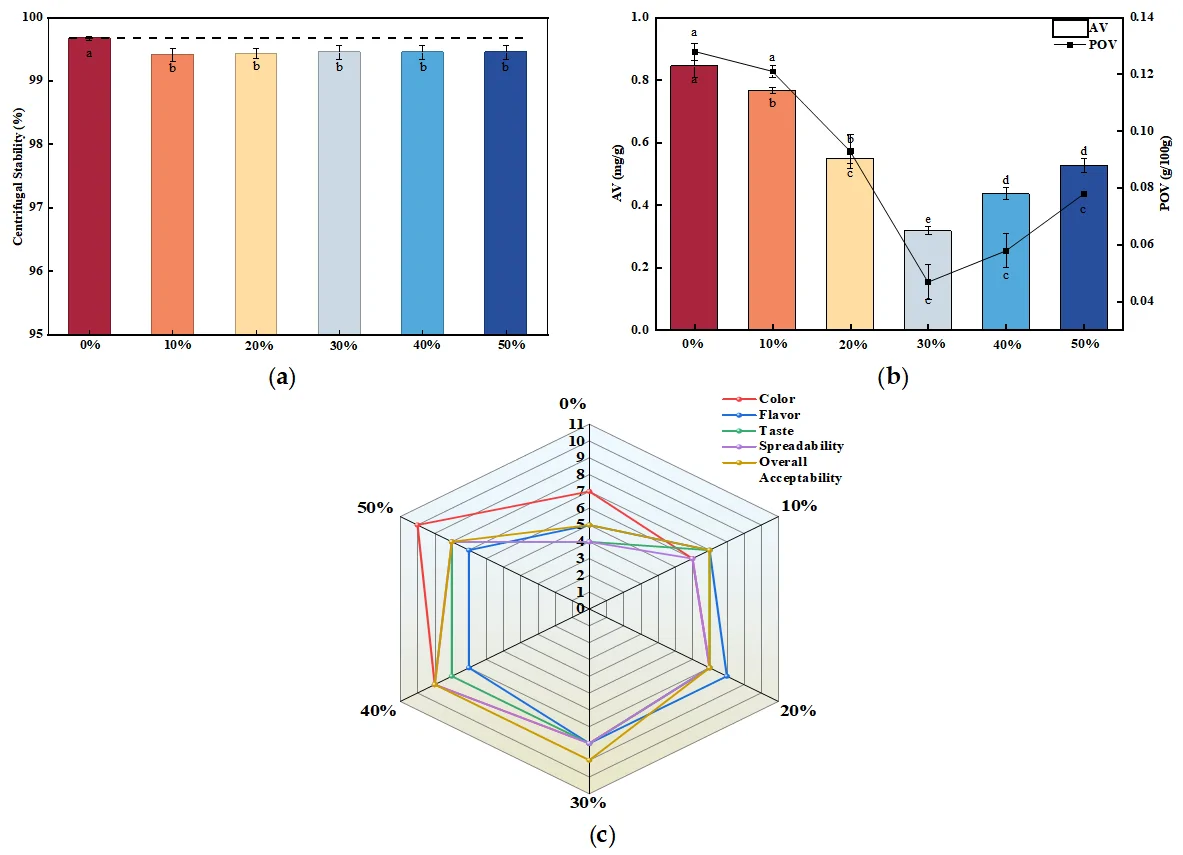
Comprehensive quality attributes of butter spreads with different CPE substitution ratios: (**a**) centrifugal oil separation rate; (**b**) oxidative stability (acid value, AV; peroxide value, POV); (**c**) sensory evaluation scores. (CPE substitution ratios were 0–50%.) Data are presented as mean ± standard deviation (*n* = 3). Different lowercase letters indicate significant differences (*p* < 0.05).

**Table 1:** Formulation of butter spread with different CPE substitution ratios.

Sample	Ingredient (g/100 g)				
	Butter	CPE	Powdered Sugar	Skimmed Milk Powder	Sterile Water
0%	60	0	12.5	12.5	15
10%	54	6			
20%	48	12			
30%	42	18			
40%	36	24			
50%	30	30			

Note: The total fat phase was fixed at 60 g/100 g (butter + CPE). The CPE substitution level is defined as the mass fraction of CPE within the total fat phase (*w*/*w*). Therefore, substitution levels of 0%, 10%, 20%, 30%, 40%, and 50% correspond to 0, 6, 12, 18, 24, and 30 g CPE per 100 g, respectively. All other ingredients remained constant across formulations.

**Table 2:** Effect of different CPE substitution ratios on the color of butter spreadable paste.

	L*	a*	b*	C*
0%	78.07 ± 2.83 ^a^	−4.21 ± 0.10 ^d^	36.01 ± 1.76 ^a^	30.43 ± 0.70 ^c^
10%	65.89 ± 3.07 ^b^	16.31 ± 1.82 ^c^	33.68 ± 0.33 ^ab^	39.54 ± 2.35 ^b^
20%	58.45 ± 1.17 ^c^	16.56 ± 0.50 ^bc^	33.05 ± 1.49 ^b^	38.92 ± 2.32 ^a^
30%	58.41 ± 1.75 ^c^	20.54 ± 1.99 ^c^	31.31 ± 1.39 ^bc^	37.54 ± 0.49 ^b^
40%	52.66 ± 2.11 ^d^	24.90 ± 2.85 ^b^	30.13 ± 0.72 ^bc^	40.13 ± 0.67 ^b^
50%	51.29 ± 0.91 ^d^	32.74 ± 5.12 ^a^	21.32 ± 0.25 ^c^	45.43 ± 0.54 ^b^

L*, lightness; a*, redness/greenness value; b*, yellowness/blueness value. Different lowercase letters in the same column indicate significant differences among groups (*p* < 0.05).

**Table 3:** Texture profile of butter spread samples with different CPE substitution ratios.

	0%	10%	20%	30%	40%	50%
Hardness (N)	0.35 ± 0.07 ^a^	0.32 ± 0.05 ^a^	0.26 ± 0.03 ^ab^	0.20 ± 0.01 ^bc^	0.19 ± 0.02 ^bc^	0.12 ± 0.01 ^c^
Chewiness (mJ)	1.08 ± 0.03 ^bc^	1.23 ± 0.09 ^a^	1.11 ± 0.02 ^b^	1.10 ± 0.05 ^b^	0.98 ± 0.02 ^cd^	0.88 ± 0.07 ^d^

Different lowercase letters in the same column indicate significant differences among groups (*p* < 0.05).

## Data Availability

The original contributions presented in the study are included in the article. Further inquiries can be directed to the corresponding author.

## Abbreviations

The following abbreviations are used in this manuscript:

WPI	Whey Protein Isolate
WalPC	Walnut Protein Concentrate
HPP	High Pressure Processing
AV	Acid Value
POV	Peroxide Value
HTST	High-Temperature Short-Time
